# Management of brain metastases: history and the present

**DOI:** 10.1186/s41016-018-0149-0

**Published:** 2019-01-18

**Authors:** Qi Liu, Xuezhi Tong, Jiangfei Wang

**Affiliations:** Department of neurosurgery, Beijing Tiantan Hospital, Capital Medical University, Fengtai District, Southern 4th Street, No.119, Beijing, 100071 China

**Keywords:** Brain metastases, Management, Surgery, Whole brain radiotherapy, Stereotactic radiosurgery, Tyrosine-kinase inhibitors, Immunotherapy

## Abstract

Brain metastases are significant causes of morbidity or mortality for patients with metastatic cancer. With the application of novel systematic therapy and improvement of overall survival, the prevalence of brain metastases is increasing. The paradigm of treatment for brain metastases evolved rapidly during the last 30 years due to the development of technology and emergence of novel therapy. Brain metastases used to be regarded as the terminal stage of cancer and left life expectancy to only 1 month. The application of whole brain radiotherapy for patients with brain metastases increased the life expectancy to 4–6 months in the 1980s. Following studies established surgical resection followed by the application of whole brain radiotherapy the standard treatment for patients with single metastasis and good systematic performance. With the development of stereotactic radiosurgery, stereotactic radiosurgery plus whole brain radiotherapy provides an alternative modality with superior neurocognitive protection at the cost of overall survival. In addition, stereotactic radiosurgery combined with whole brain radiotherapy may offer a promising modality for patients with numerous multiple brain metastases who are not eligible for surgical resection. With the advancing understanding of molecular pathway and biological behavior of oncogenesis and tumor metastasis, novel targeted therapy including tyrosine-kinase inhibitors and immunotherapy are applied to brain metastases. Clinical trials had revealed the efficacy of targeted therapy. Furthermore, the combination of targeted therapy and radiotherapy or chemotherapy is the highlight of current investigation. Advancement in this area may further change the treatment paradigm and offer better modality for patients who are not suitable for surgical resection or radiosurgery.

## Introduction

Brain metastasis is always an indication of poor prognosis, with short overall survival, progression-free survival, and neurological deterioration [1]. The incidence of brain metastases from unselected patients with different kinds of tumors ranges from 8% to 10% [2]. The most common type of primary tumor is lung cancer, which accounts for approximately 20% of the brain metastases, followed by breast, melanoma, renal, and colorectal cancer. Among these kinds of cancers, melanoma is most likely to metastasize to the brain. Autopsy reported that nearly 75% of the patients died of melanoma-developed brain metastases [3]. While among patients with non-small cell lung cancer, around 20%–40% will develop brain metastasis at some point. Most studies reported that there is no difference in incidence between male and female, despite a few exceptions [4, 5]. The prevalence of brain metastases is increasing due to novel therapeutics resulting in improved survival, the aging population, and advancing imaging techniques [6, 7].

The main goal of treatment of brain metastases is to achieve local control of the metastatic lesion, to improve life quality and to prevent death from neurological disease [6]. The treatment strategy should be selected cautiously dependent on the number of tumor size, general systematic performance, neurological function, type of tumor, and so on. This article reviewed the evolvement of the changing paradigm of modality method for patients with brain metastases based on the evidence from clinical trials and wish to provide and insight for the treatment method.

## Conventional therapy

Convention therapy including surgical resection, whole brain radiotherapy (WBRT), and stereotactic radiosurgery (SRS) and the combination of them were introduced to the treatment of brain metastases since 1980s. The combination of these modalities remains the preferred treatment for selected patients, especially for those with good systematic performance. The advancement in these methods is continuing to extend their application.

### Surgical resection

Surgical resection is indicated for patients with solitude brain metastasis in accessible location or with tumor in large size causing brain edema or hydrocephalus. Patients with controlled disease and good condition are preferred to surgical resection.

In the 1980s, several studies revealed benefits of surgical resection for patients with brain metastases [8–10]. Afterwards, the results of several randomized clinical trials published in the 1990s confirmed the superiority of combined surgical resection and WBRT compared against the WBRT alone for patients with solitude brain metastasis and good functional conditions [11, 12]. In a randomized clinical trial [11], a total of 48 patients were randomly assigned to surgical plus WBRT group and radiation group. Surgical resection combined with WBRT resulted in lower local recurrence rate, longer overall survival, and progression-free survival. The patients with combination modality also maintain a longer time of good quality of life (Karnofsky score ≥ 70). However, surgical resection had no influence on the occurrence of the distant brain metastases and leptomeningeal metastases.

While surgical resection exhibited to be beneficial for patients with one metastasis and good systemic condition, there is not enough robust evidence to evaluate the role of surgical resection for multiple brain metastases [13]. Few retrospective studies showed the efficacy of resection of multiple brain metastases [14, 15]. In the retrospective review conducted by MD Anderson Cancer [14], the patients with multiple brain metastases were assigned into two groups. Twenty six of the patients underwent resection of all the metastases, while 30 of the patients left one or more lesions unresected. Another group of 26 patients with single metastasis who underwent resection was set to match the groups. The patients in whom all the lesions were resected had better prognosis as regards to overall survival (14 months *vs* 6 months). There is no difference between the survival of patients with all lesions resected and patients in the additional group.

As regards to the surgical resection of the recurrent brain metastases, previous studies revealed that reoperation resulted in significant function improvement, better quality of life, and protracted overall survival [16, 17]. The investigation of Al-Zabin et al. revealed that [16], after reoperation, 66.6% of patients presented with ameliorated neurological impairment and 50% regained normal function. The median Karnofsky index increased significantly from 80 to 100 after the second surgery. Arbit et al. [17] found that for patients with recurrent brain metastases, those who underwent reoperation had longer median survival time compared to those who did not. This study indicated that surgery is effective in prolonging the survival of patients with brain metastases. However, more convinced evidence from prospective clinical trial is needed to confirm those findings.

Advantages of surgical resection include providing histological diagnosis, avoiding long-term use of steroid, immediate amelioration of mass effect, and neurologic deficit [1]. The rate of complication varies from different trial reports. Application of novel technologies and new adjuncts of surgery such as intraoperative image guidance and intraoperative monitoring can help reduce the risk of resection and increase the extent of tumor removal [6].

### Whole brain radiation therapy

The life expectancy used to be only 1 month for patients with brain metastases, and the use of steroids protracted the overall survival to 2 months [18]. WBRT is always used as an adjuvant therapy after surgical resection or for multiple metastases which is not suitable for surgical resection. The use of whole brain therapy was reported to further lengthen the median survival time to 4–6 months [9].

In the 1970s, the Radiation Therapy Oncology Group (RTOG) conducted a series of clinical trials to examine the efficacy of radiation therapy and explore the appropriate fractionation schemes. Several randomized controlled trials (RCTs) established the WBRT as an important adjunct therapy of surgical resection and SRS, which will be discussed later in this article.

The toxicity of WBRT remained the concern of clinical treatment. Brown et al. launched a randomized, double-blinded, placebo-controlled trial to evaluate whether memantine is efficient in palliating the neurological decline [19]. They turned out to find that the use of memantine can delay the occurrence of cognitive decline. Although the rate of cognitive decline is not significantly different from the controlled group at 24 weeks, that may be a result of exodus of eligible patients and low statistical power. New approach was also designed to prevent neurological decline. Based on the rationale that hippocampal stem-cell injury during WBRT may contribute to the memory decline, hippocampal avoidance WBRT (HA-WBRT) was applied to limit the injury to hippocampus. In the phase 2 clinical trial, the Hopkins Verbal Learning Test-Revised Delayed Recall (HVLT-R DR) was used to evaluate the efficacy. Mean relative decline in HVLT-R DR is only 7% from baseline in 4 months, which is significantly lower than that of a historical control group [20]. These trials put forward novel method for the prevention of neurological decline and may resurrect the status of WBRT in brain metastases.

As regards to the influence of types of tumor, the result of Sundstrom JT’s study showed no significant difference of the overall survival between patients with classic radioresistant tumor and the rest [21]. However, further study with more robust evidence is needed to confirm that result.

### Stereotactic radiosurgery (SRS)

In 2006, the American Association of Neurological Surgeons (AANS) and American Society for Therapeutic Radiology and Oncology (ASTRO) defined stereotactic radiosurgery as “a distinct neurosurgical discipline that utilizes externally generated ionizing radiation to inactivate or eradicate defined targets in the head or spine without the need to make an incision.” SRS is usually performed in a single session, up to a maximum of five sessions, using accelerator under the guidance of real-time imaging. Since its introduction, SRS has been evolving from an investigational concept into one of the mainstream neurosurgical modality for various brain disorders [22].

The SRS was first evaluated for its efficacy by combing with WBRT to compare with WBRT alone. Two randomized clinical trial confirmed its benefit in overall survival and local control for patients with up to four brain metastases [23, 24]. Andrew et al. [24] enrolled 333 patients from 55 institutions, with 167 who underwent WBRT plus SRS and 164 who allocated WBRT alone. The SRS plus WBRT group is more likely to have stable or improved Karnofsky performance status (KPS) score at 6 months. And there is an advantage in overall survival for patients with a single brain metastasis to accept WBRT followed by SRS. The investigation by Kondziolka et al. revealed a significant decrease in the rate of local failure and extension of overall survival and interval before local failure for patients with two to four brain metastases [23].

With the confirmation of the benefit of SRS, researches were designed to look into the necessity of WBRT by comparing SRS plus WBRT and WBRT alone for patients with limited number of brain metastases (< 4). In the randomized controlled trial (RCT) conducted by Aoyama et al., 132 patients were assigned to either SRS plus WBRT or SRS alone [25]. While there is no difference in the overall survival or toxic effects between the two groups, patients who underwent WBRT alone had a higher incidence of relapse and are more prone to salvage treatment. The following clinical trials further investigate the toxicity of additional WBRT by setting the primary end point as an impaired neurocognitive function [26, 27]. The patients with combined therapy had a lower rate of recurrence at the expense of higher rate of decline in memory and learning. The researchers thus recommend an initial treatment of combined SRS and WBRT and close clinical monitoring for better preservation of cognitive function. The study by Kocher et al. reached the similar conclusion [27]. In a word, the combination of SRS and WBRT decreases the risk of relapse but leads to a higher rate of decline in neurological function. The modality should be selected cautiously dependent on the systematic performance and patients’ willingness and close clinical monitoring is necessary.

Previous studies limit the eligibility of patients as having no more than four brain metastases. With the development of technology and the application of SRS for more lesions in one session, further studies investigated the efficacy of SRS for patients with more lesions. The prospective observational study conducted by Yamomoto et al. revealed that SRS alone without WBRT in patients with five or more brain metastases is non-inferior to that in patients with two to four lesions in terms of overall survival and rate of treatment-related adverse events [28]. The following analysis of the same study gives insight of the safety of SRS alone for the target patients [29]. Mini Mental State Examination (MMSE) score was applied to evaluate the neurological function. There is no difference as regards to MMSE score maintenance or post-SRS complication rate between patients with single, two to four lesions and five or more patients. It may help consist the landscape of SRS treating patients with multiple brain metastases.

## Combination of conventional therapy

Combination of conventional therapy including surgery and radiotherapy is the current standard modality for patients with brain metastases, due to the robust evidence showing its efficacy and moderate complication. Several prospective studies as well as systematic review compared the benefit of these modalities and recommended management strategy has been designed for patients with brain metastases [6, 13, 30].

### Surgical resection plus WBRT

Several clinical trials investigated the superiority of surgical resection plus WBRT against surgery or WBRT alone.

#### Surgical resection plus WBRT versus WBRT alone

Two prospective randomized trials compared the surgical resection plus WBRT versus WBRT alone [12, 31]. Vecht et al. [12] enrolled 63 eligible patients with a single brain metastasis in their clinical trial, who were randomized assigned with surgery plus WBRT or WBRT alone. All of the patients were prescribed with the same schedule of WBRT. The combined therapy led to longer overall survival (10 months *vs* 6 months) and functionally independent survival. Patients with combined modality also benefited from immediate functional improvement. However, another clinical trial failed to demonstrate the benefit of additional surgery to WBRT. There is no significant difference between the overall survival, mortality, and morbidity rate or cause of death of the patients in two groups.

A retrospective study conducted by Rades et al. [32] evaluated 195 patients who either underwent surgery plus WBRT or WBRT alone. The result showed that additional resection to WBRT in patients with solitary lesion improved overall survival. The median overall survival was 6 months for the patients treated with WBRT alone and 11.5 months for those with surgery plus WBRT. Univariate analysis revealed that better local control is associated with section. However, the combined therapy failed to prevent the development of new brain metastases. Another retrospective study conducted by RTOG also showed improved survival in elected patients [33].

#### Surgical resection plus WBRT versus surgical resection alone

In 1998, the result of a randomized clinical trial established the status of postoperative WBRT as the standard care for patients with brain metastases [34]. A total of 95 patients with a single brain metastasis were enrolled and randomly assigned to two groups. Postoperative WBRT decreases the rate of both local and distant recurrence, and the incidence of death from neurological death. However, there is no difference between the overall survival among the two groups of patients.

Another three retrospective cohort studies also showed superiority of combined surgical resection and WBRT against surgery alone for patients with brain metastases from lung cancer and melanoma [35–37].

### SRS plus WBRT

#### Surgical resection plus WBRT versus SRS plus WBRT

Several retrospective studies compared the efficacy and toxicity of these two modalities [38, 39]. The investigation of Schoggl et al. revealed that the overall survival of the patients treated by SRS plus WBRT is comparable to that of patients treated by surgical resection plus WBRT [38]. In addition, patients who underwent SRS had lower rate of local recurrence. Thus, the author recommended SRS plus WBRT rather than surgical resection plus for patients except for cases of lesions larger than 3 cm and those with mass effect. A matched pair analysis expressly compared the two modalities for patients with one to three brain metastases [39]. SRS plus WBRT presented better overall survival and lower local recurrence rate. The study concluded that the modality of SRS plus WBRT is at least as effective as an operation plus WBRT.

However, there is no evidence to advocate the use of SRS for patients with a lesion > 3 cm or for those with four or more brain metastases.

## Targeted therapy

With the advanced understanding of molecular pathways that mediate oncogenesis and brain metastasis, more preclinical and clinical research investigated the clinical use of targeted therapy for brain metastases [40]. While conventional therapies mainly target the patients with good systematic condition, targeted therapy may cast a light for patients who are not eligible for surgery of radiotherapy.

### Tyrosine-kinase inhibitor (TKIs)

The discovery of epidermal growth factor receptor (EGFR) mutation in non-small cell lung cancer and other types of cancers and the application of EGFR inhibitor have launched a new era of personalized medicine in patients with advanced non-small cell lung carcinoma (NSCLC) [41]. About 25%–40% of patients with NSCLC develop brain metastases during the course of the disease [42]. In the past decade, a clinical trial has revealed a promising response rate of EGFR inhibitors for NSCLC patients with brain metastases.

Traditional chemotherapeutic agents failed to penetrate the BBB due to its large molecular weight. Similarly, the cerebrospinal fluid (CSF) penetration of the first- and second-generation TKI is low, with average CSF penetration rates of < 1%, 1%–3%, and 3%–6% reported for afatinib, gefitinib, and erlotinib, respectively. However, the penetration of gefitinib is enhanced in patients with brain metastases, possibly due to the tumor-induced BBB disruption [43].

Nevertheless, EGFR TKIs has demonstrated efficacy for patients with brain metastases. A meta-analysis revealed that EGFR TKIs combined with SRS or WBRT lead to improved response rate, prolonged progression-free survival (PFS), and overall survival [44]. The result of a phase 2 study showed that gefitinib alone without radiotherapy can lead to a favorable response rate, PFS, and overall survival [45]. In addition, patients with exon 19 deletion exhibited a better outcome. A phase 2 trial of erlotinib plus concurrent WBRT for patients with brain metastases was conducted by Welsh et al. [46]. The overall response rate was 86%, and no increase in neurotoxicity is detected.

The major challenge TKIs encountered is acquired drug resistance, which occurs in a majority of patients treated with the first and second generation of TKIs [47]. A new generation of TKIs including AZD3759 has been designed to overcome the challenge and improve the central nervous system penetration [48]. Further study may investigate the efficacy the novel drug for brain metastases.

### Immunotherapy

#### Cytotoxic T-lymphocyte-associated protein 4 (CTLA-4) inhibitor

CTLA-4 is a protein receptor functioning as a check point in the T cells to downregulate its immune response when bounded to CD80 or CD86. It is commonly expressed in the regulatory T cells and upregulated in the activated conventional T cells, which is a notable phenomenon in cancers [49]. Ipilimumab was the first CTLA-4 inhibitor approved by the US Food and Drug Administration (FDA) in 2011 for the treatment of unresectable or metastatic melanoma.

An open-label, phase 2 trial investigates the efficacy and safety of ipilimumab alone in patients with melanoma and brain metastases. The central nervous system (CNS) response rate is 16% in asymptomatic patients and 5% in symptomatic patients. The overall survival is 2.7 months for asymptomatic patients and only 1.3 months for symptomatic patients.

In an open-label, single-arm phase 2 trial, 86 eligible patients were treated with fotemustine plus ipilimumab [50]. The disease control rate for patients with brain metastases was 30% (11.9%–54.3%). Median PFS of patients with brain metastasis is 4.5 months, which is shorter than that of all patients. Absolute lymphocyte count increases in patients achieving disease control. However, it is not significant compared to that of patients with progression disease. Treatment-related adverse events were generally manageable, with 24 patients suffering from grade 3 or 4 adverse events.

#### PD-1/PD-L1 inhibitor

Programmed cell death protein 1 (PD-1) is another important check point expressed in the T cells that negatively regulate the immune response when bounded to its ligands programmed death-ligand 1 (PD-L1) or programmed death-ligand 2 (PD-L2). It works by promoting the apoptosis of antigen-specific T cells and suppressing the apoptosis of the regulatory T cells [51, 52].

Approved PD-1 inhibitors include nivolumab and pembrolizumab, while approved PD-L1 inhibitors include atezolizumab, avelumab, and durvalumab. Several clinical trials are ongoing to evaluate their efficacy and safety on melanoma, NSCLS, renal cell carcinoma (RCC), and so on.

A phase II clinical trial investigated pembrolizumab for patients with melanoma of NSCLC and untreated brain metastases (NCT02085070) [53]. A total of 36 patients was enrolled, 18 with melanoma and 18 with NSCLC. All of the patients were free of neurological symptoms or the need for corticosteroids, with the size of brain metastases between 5 and 20 mm. Tumor PD-L1 positivity is required for NSCLC patients. Patients were prescribed with pembrolizumab 10 mg/kg every 2 weeks until progression. The CNS response rate (partial response plus complete response) was 22% and 33% among patients with melanoma and NSCLC, respectively. CNS response time is durable ranging from 4 to 10 months for melanoma and 3 to 7 months for NSCLC. None of the patients of NSCLC with a Kirsten rat sarcoma viral oncogene (KRAS) mutation had a CNS response. Pembrolizumab is well tolerated among the patients. The majority of the treatment-related adverse events are of grades 1–2. Treatment-related grade 3–4 adverse events were rare, including gastrointestinal symptoms, pneumonitis, and constitutional symptoms. One patient of melanoma suffered from cognitive dysfunction due to pembrolizumab, disease, or both. Previous studies revealed that pembrolizumab, compared to CTLA-4 inhibitor ipilimumab, prolonged the progression-free survival and overall survival in patients with advanced melanoma and had a lower rate of serious adverse event [54, 55]. However, these studies excluded patients with brain metastases or did not compare the outcomes between the patients with brain metastases and those without. To our knowledge, the survey described above was the first to evaluate the efficacy of PD-1 axis inhibitor in the CNS melanoma or NSCLC patients expressly.

#### Combined CTLA-4 inhibitor and PD-1/PD-L1 inhibitor

An open-label, multicenter, phase study investigated the efficacy and safety of combined nivolumab and ipilimumab for patients with brain metastases [56]. Ninety-four patients who had histologically confirmed malignant melanoma with brain metastases and Eastern Cooperative Oncology Group (ECOG) performance status of 0 or 1 were eligible for the study. The primary end point was the rate of intracranial benefit, defined as the percentage of patients with complete response, partial response, and stable disease for at least 6 months. Among the 94 patients, 57% (95% CI, 47%–68%) patients had intracranial clinical benefit, with the complete response rate of 26%, the partial response rate of 30%, and the stable disease rate for at least 6 months of 2%. The rate of extracranial clinical benefit was 56% (95% CI, 46%–47%), concordant with that of intracranial benefit. This modality also presented rapid response (with median response time of 2.1 months, ranging from 1.1 to 15 months) and appreciable duration time. The rate of grade 3 or 4 treatment-related adverse event was 55%, the most common of which were an increase of alanine aminotransferase or aspartate aminotransferase. CNS adverse events occurred in 36% of the patients, and seven patients presented grade 3 or 4 CNS adverse events. The response rate is higher than ipilimumab alone (24%) [57] or pembrolizumab alone (22%) [53] and similar to combined ipilimumab and fotemustine (50%) [50].

## Combined immunotherapy and radiotherapy

The rationale behind the combination of the immunotherapy was derived from abscopal effect. Abscopal effect refers to the phenomenon that treatment of the local tumor leads to the concurrent shrinkage of the metastatic tumor at distant sites. The hypothesis is that radiation leads to the death of tumor and thus the liberation of tumor-associated antigens (TAAs) [58]. These antigens can be recognized and processed by the antigen-presenting cells. Then, cytotoxic T cells can be primed to target the tumor cells at distant areas.

Combining radiotherapy with immunotherapy provides opportunities for boosting abscopal response rate and further use of radiotherapy for local and metastatic tumors [58].

Several studies had been conducted to investigate the efficacy and safety of the combination of immunotherapy and radiotherapy for brain metastases.

One study proved that the concurrent use of immunotherapy and SRS resulted in appreciable reduction in lesion volume [59]. A total of 75 patients with 566 melanoma brain metastases was included. The reduction in lesion was significantly greater for the concurrent group than the non-concurrent group at 1.5 months, 3 months, and 6 months. The overall survival of patients with concurrent modality is longer than that of patients who received SRS only. In addition, the reduction of the lesion in the modality with PD-1 inhibitor was greater than that of the modality with CTLA-4 inhibitor.

However, it should be noted that another retrospective study showed no significant difference between the survival of patients who underwent SRS alone and patients with concurrent ipilimumab and SRS [60].

A phase 1 study was designed to determine the safe dose of ipilimumab with SRS or WBRT for the treatment of melanoma with brain metastasis [61]. The median PFS time for patients who had undergone ipilimumab with WBRT and ipilimumab with SRS is 2.53 months and 2.45 months respectively. The PFS was 2.5 months in patients treated with ipilimumab and WBRT and 2.1 months in patients treated with ipilimumab and SRS. There is no significant difference between the two groups. The modality was well tolerated, with a total of 21 grade 1 to 2 neurotoxic effects and one grade 3 neurotoxic event.

Several trials are ongoing to investigate the efficacy, safety, proper timing of combination of radiotherapy, and immunotherapy [62]. The results of these studies will help evaluate the status of this therapy for brain metastases.

## Conclusions

The paradigm changed rapidly in the treatment of brain metastases. The selection of modality is of vital importance for the benefit of the patients. Surgical resection plus WBRT is preferred for patients with single lesion and good condition. Surgery can also lead to immediate remission of the clinical neurosurgical symptoms. SRS plus WBRT provided better neurological protection despite the shortening of overall survival. Special WBRT or adjunct therapy can be applied to decrease the rate of neurocognitive decline. The use of novel targeted therapy may put forward a new method for the patients with brain metastases, especially those with numerous multiple brain metastases. The type of primary tumor and the mutation should guide the use. Further prospective clinical trials are needed to evaluate the efficacy and safety.

## Abbreviations

AANS: American Association of Neurological Surgeons
ASTRO: American Society for Therapeutic Radiology and Oncology
CNS: Central nervous system
CSF: Cerebrospinal fluid
ECOG: Eastern Cooperative Oncology Group
EGFR: Epidermal growth factor receptor
FDA: Food and Drug Administration
HA-WBRT: Hippocampal avoidance WBRT
HVLT-R DR: Hopkins Verbal Learning Test-Revised Delayed Recall
KPS: Karnofsky performance status
KRAS: Kirsten rat sarcoma viral oncogene
MMSE: Mini Mental State Examination
NSCLC: Non-small cell lung carcinoma
PD-1: Programmed cell death protein 1
PD-L1: Programmed death-ligand 1
PD-L2: Programmed death-ligand 2
PFS: Progression-free survival
RCC: Renal cell carcinoma
RCT: Randomized controlled trial
RTOG: Radiation Therapy Oncology Group
SRS: Stereotactic radiosurgery
TKIs: Tyrosine-kinase inhibitors
WBRT: Whole brain radiotherapy
